# VNUT/SLC17A9, a vesicular nucleotide transporter, regulates osteoblast differentiation

**DOI:** 10.1002/2211-5463.12918

**Published:** 2020-07-12

**Authors:** Asako Inoue, Kayoko Nakao‐Kuroishi, Kaori Kometani‐Gunjigake, Masahiro Mizuhara, Tomohiko Shirakawa, Misa Ito‐Sago, Kazuma Yasuda, Mitsushiro Nakatomi, Takuma Matsubara, Yukiyo Tada‐Shigeyama, Kazumasa Morikawa, Shoichiro Kokabu, Tatsuo Kawamoto

**Affiliations:** ^1^ Division of Orofacial Functions and Orthodontics Department of Health Improvement Kyushu Dental University Kitakyushu‐shi Japan; ^2^ Division of Anatomy Department of Health Improvement Kyushu Dental University Kitakyushu‐shi Japan; ^3^ Division of Molecular Signaling and Biochemistry Department of Health Improvement Kyushu Dental University Kitakyushu‐shi Japan; ^4^ Division of Dental Anesthesiology Department of Science of Physical Functions Kyushu Dental University Kitakyushu‐shi Japan; ^5^ Division of Pediatric and Special Care Dentistry Department of Developmental Oral Health Science School of Dentistry Iwate Medical University Morioka Japan

**Keywords:** ATP, compressive force, osteoblast, osteoblast differentiation, P2 receptor, VNUT

## Abstract

Osteoblasts release adenosine triphosphate (ATP) out of the cell following mechanical stress. Although it is well established that extracellular ATP affects bone metabolism via P2 receptors [such as purinergic receptor P2X7 (P2X7R) and purinergic receptor P2Y2 (P2Y2R)], the mechanism of ATP release from osteoblasts remains unknown. Recently, a vesicular nucleotide transporter [VNUT, solute carrier family 17 member 9 (SLC17A9)] that preserves ATP in vesicles has been identified. The purpose of this study was to elucidate the role of VNUT in osteoblast bone metabolism. mRNA and protein expression of VNUT were confirmed in mouse bone and in osteoblasts by quantitative real‐time PCR (qPCR) and immunohistochemistry. Next, when compressive force was applied to MC3T3‐E1 cells by centrifugation, the expression of *Slc17a9*, *P2x7r*, and *P2y2r* was increased concomitant with an increase in extracellular ATP levels. Furthermore, compressive force decreased the osteoblast differentiation capacity of MC3T3‐E1 cells. shRNA knockdown of *Slc17a9* in MC3T3‐E1 cells reduced levels of extracellular ATP and also led to increased osteoblast differentiation after the application of compressive force as assessed by qPCR analysis of osteoblast markers such as Runx2, Osterix, and alkaline phosphatase (ALP) as well as ALP activity. Consistent with these observations, knockdown of *P2x7r* or *P2y2r* by siRNA partially rescued the downregulation of osteoblast differentiation markers, caused by mechanical loading. In conclusion, our results demonstrate that VNUT is expressed in osteoblasts and that VNUT inhibits osteoblast differentiation in response to compressive force by mechanisms related to ATP release and P2X7R and/or P2Y2R activity.

## Graphical abstract

In this study, we show that VNUT (SLC17A9), a vesicular nucleotide transporter, regulates osteoblast differentiation in response to mechanical loading. Mechanical loading promotes adenosine triphosphate (ATP) exocytosis via VNUT. Extracellular ATP subsequently inhibits osteoblast differentiation via activation of P2X7 and/or P2Y2 receptors. Thus, VNUT modulation of purinergic signaling contributes to the regulation of bone metabolism by mechanical loading.

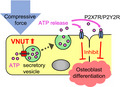

**Abbreviations**
ALP: alkaline phosphatase
ATP: adenosine triphosphate
P2X1R: purinergic receptor P2X1
P2X7R: purinergic receptor P2X7
P2Y2R: purinergic receptor P2Y2
qPCR: quantitative real‐time PCR
SLC17A9: solute carrier family 17 member 9
VNUT: vesicular nucleotide transporter

Bone homeostasis is maintained by the balanced activity of bone‐forming osteoblasts and bone‐resorbing osteoclasts. Mechanical stress and loading are key factors for the regulation of bone homeostasis [1]. Osteoblasts can sense mechanical stress and respond either directly or via coordinated crosstalk with osteoclasts [2, 3, 4, 5, 6]. Long periods of inadequate mechanical loading, for example, during continued bed rest or the microgravitational environment of space flight, result in bone loss [7, 8]. In orthodontic treatment, asymmetric mechanical loading to alveolar bone is used as a tool to move and adjust the position of teeth [9, 10]. Thus, understanding the effects and mechanisms of mechanical loading on bone cells is important for successful treatment of unloading‐related osteoporosis, effective orthodontic treatment, orthopedic surgery, and physical therapy.

Mechanical loading induces multiple cellular events in osteoblasts. A major effect of mechanical loading on osteoblasts is the efflux of adenosine triphosphate (ATP) into the extracellular space [11, 12, 13, 14]. Extracellular ATP can then subsequently act as a signaling molecule in an autocrine or paracrine manner, to activate members of the P2 class of extracellular nucleotide receptors. Purinergic receptor P2X1 (P2X1R), purinergic receptor P2X7 (P2X7R), and purinergic receptor P2Y2 (P2Y2R) have been reported to be involved in bone remodeling [11, 12, 13, 14, 15, 16, 17, 18]. Variations in P2 receptor subtype expression and nucleotide ligand responsiveness, however, mean that the net effect of extracellular nucleotides on osteoblast differentiation is unclear. For example, whereas intermittent compressive force applied to human mandibular bone‐derived osteoblast cells releases ATP, activates P2X1R, and promotes osteoblast differentiation [14], the activation of P2X7R by the selective agonist BzATP suppresses the differentiation of primary human osteoblasts [15]. It has also been reported that low concentrations of ATP and UTP strongly inhibit osteogenesis in osteoblasts via the P2Y2R [16]. Furthermore, extracellular ATP can be metabolized into ADP and adenosine by the action of ectonucleotidases and ATP can also act as a source of phosphate for osteoblast mineralization. Despite the large body of evidence that extracellular ATP affects osteoblast activity, little is known about the mechanism by which ATP is secreted.

Solute carrier family 17 member 9 (SLC17A9) was recently identified as a vesicular nucleotide transporter (VNUT), essential for ATP secretion from adrenal PC12 cells [19]. ATP can be released from cells via multiple mechanisms including through pannexin or connexin hemichannels [20], maxi‐anion channels [21], P2X7R [22], and vesicular exocytosis [23]. VNUT localizes to secretory vesicle membranes and is involved in the efflux of ATP via vesicular exocytosis. That is, VNUT transports ATP from the cytosol into the secretory vesicle from where ATP can subsequently be exocytosed upon stimulation. So far, VNUT expression has been reported in various cells such as odontoblasts, periodontal ligament cells, limbal epithelial cells, conjunctival epithelial cells, and gastric epithelial cells [24, 25, 26, 27, 28]. Given the importance of extracellular ATP in osteoblast cell function, we hypothesized that VNUT may play a role in osteoblast activity.

Here, we report that VNUT is expressed in osteoblasts where it plays a role in ATP secretion as well as osteoblast differentiation.

## Materials and methods

### Cell culture

MC3T3‐E1 cells, a mouse calvarial osteoblast cell line, were obtained from Riken Bio Resource Center (Tsukuba, Japan). MC3T3‐E1 cells were maintained in α‐minimal essential medium (12571‐063; Gibco‐BRL, Grand, Island, NY, USA) supplemented with 10% FBS (Biosera Nuaillé, France) and 100 U·mL^−1^ penicillin–streptomycin (FUJIFILM Wako Pure Chemical Corporation, Osaka, Japan). Osteoblast differentiation was induced by culturing cells in an osteogenic medium containing 50 μg·mL^−1^ ascorbic acid (FUJIFILM Wako Pure Chemical Corporation) and 10 mm β‐glycerophosphate (Sigma‐Aldrich, St. Louis, MO, USA) for 7 days. Cells were treated with 1, or 10 μm clodronate (Sigma‐Aldrich). Ionomycin (AdipoGen Life Sciences, San Diego, CA, USA) treatments were performed at 1 μm. Brefeldin A (Cayman Chemical, Ann Arbor, MI, USA) was used at 10 μm. HEK293T cells (Takara Bio, Shiga, Japan) were cultured in Dulbecco's modified Eagle's medium (DMEM) (12320‐032; Gibco‐BRL) supplemented with 10% FBS (Biosera Nuaillé) and 100 U·mL^−1^ penicillin–streptomycin (FUJIFILM Wako Pure Chemical Corporation).

### Application of mechanical loading

Compressive loading by centrifugation was previously described [29]. Briefly, cells were seeded at a density of 3 × 10^5^ cells per well in 12‐well plates. Medium was changed into 4‐(2‐hydroxyethyl)‐1‐piperazineethanesulfonic acid (HEPES)‐buffered DMEM without bicarbonate (12320‐032; Gibco‐BRL), supplemented with 18 µm of hydrochloric acid (Nacalai Tesque Inc., Kyoto, Japan) to stabilize pH and then cells placed in an incubator (Yamato Scientific Co. Ltd., Tokyo, Japan) at 37 °C for 1 h. The plates were subsequently centrifuged at 4.4 × 10^−2^ N·cm^−2^ (5.0 g·cm^−2^) or 8.8 × 10^−2^ N·cm^−2^ (9.0 g·cm^−2^) using a PlateSpin II centrifuge (Kubota Corp., Tokyo, Japan) in the incubator at 37 °C for 12 h. Compressive force by weights was also previously described [30]. Briefly, a thin glass plate was placed unto the cells and a force of 8.8 × 10^−2^ N·cm^−2^ was then applied for 12 h by placing a load unto the glass plate.

### Measurement of extracellular ATP

Extracellular ATP was measured in medium samples collected from cells using an ATP assay kit (TOYO B‐Net, Tokyo, Japan) according to manufacturer's instructions [24] and an AB‐2270 luminometer (ATTO Corp., Tokyo, Japan).

### RNA extraction, reverse transcription, and quantitative real‐time PCR

Heart, liver, kidney, skeletal muscle, white adipose tissue, testis, tongue, bone, and small intestine were obtained from 10‐week‐old male C57BL/6J mice. Total RNA was isolated from each organ using a RNAqueous Kit (Ambion; Life Technologies, Austin, TX, USA) and then reverse‐transcribed into cDNA using SuperScript VILO Master Mix (Thermo Fisher Scientific, Waltham, MA, USA). cDNA was amplified by quantitative real‐time PCR (qPCR) using specific primers for *Slc17a9* (forward, AATCCTCACCGGTCTGCTC; reverse, AAAGGCTCTCTCGCTCTCCT: NM_183161), *Collagen 1a1* (forward, GGTATGCTTGATCTGTATCTG; reverse, TCTTCTGAGTTTGGTGATACG: NM_007742), *Runx2* (forward, TTCAACGATCTGAGATTTGTGGG; reverse, GGATGAGGAATGCGCCCTA: NM_001146038), *Osterix* (forward, AGAGATCTGAGCTGGGTAGAGG; reverse, AAGAGAGCCTGGCAAGAGG: NM_130458), *Alkaline phosphatase* (*Alp*) (forward, CGGGACTGGTACTCGGATAA; reverse, ATTCCACGTCGGTTCTGTTC: NM_007431), *P2x1r* (forward, CATGGGGACAGCTCCTTTGT; reverse, GAGTGCAGCCACTGTCATCT: NM_008771), *P2x7r* (forward, TGCAGCTGGAACGATGTCTTG; reverse, CGCTGGTACAGCTTATCGCTCA: NM_011027), *P2y2r* (forward, TCAAACCGGCTTATGGGACC; reverse, TCAAACCGGCTTATGGGACC: NM_002564), or *β‐actin* (forward, AAGGCCAACCGTGAAAAGAT; reverse, GTGGTACGACCAGAGGCATAC: NM_007393), and PowerUp SYBR Green Master Mix (Thermo Fisher Scientific) with a QuantStudio 3 thermal cycler (Thermo Fisher Scientific). The following cycling parameters were used: 40 cycles of 15 s denaturation at 95 °C and 60 s annealing/extension at 60 °C. Values were normalized to β‐actin using the $2^{‐\Delta \Delta C_{t}}$ method [31].

### Immunohistochemistry

Animal experiments were reviewed and approved by the Kyushu Dental University Animal Care and Use Committee (#18‐003). Wild‐type mouse pups on a C57BL/6J genetic background were sampled at postnatal day 5. Calvaria bones were fixed with 4% paraformaldehyde (Merck KGaA, Darmstadt, Germany) in PBS, dehydrated through an ethanol series, embedded in paraffin, and cut into 4‐µm frontal sections [32]. Immunostaining was performed with anti‐VNUT rabbit polyclonal antibody (1 : 50 dilution). This antibody against rat or mouse VNUT was generated in rabbits using synthetic peptides corresponding to residues 5–19, RSSLMQPIPEETRKT [24] or anti‐VNUT guinea pig polyclonal antibody (1 : 50 dilution, ABN83; Sigma‐Aldrich), biotinylated anti‐rabbit IgG (1 : 400 dilution, PK‐6101; Vector Laboratories, Burlingame, CA, USA) or anti‐Guinea pig IgG HRP (1 : 100 dilution, NB7398; Novus Biologicals, Centennial, CO, USA), and the Vectastain Elite ABC kit (1 : 50 dilution; Vector Laboratories), Sigmafast 3,3′‐diaminobenzidine (DAB) tablets (Sigma‐Aldrich) were used for visualization of reaction products. Immunostained sections were counterstained with diluted hematoxylin. As a negative control for anti‐VNUT rabbit polyclonal antibody, antibody was preadsorbed with the antigenic peptide by mixing with 10 µg·µL^−1^ peptide for 60 min at room temperature [24].

### Plasmid and transfection

Murine *Slc17a9* (NM_183161) was obtained by standard PCR cloning from mouse white adipose tissue cDNA using PrimeSTAR HS DNA polymerase (TaKaRa, Otsu, Japan) and subcloned into pcDNA3.1His‐V5 (Thermo Fisher Scientific). Cells were transfected with *Slc17a9* plasmid using Lipofectamine 2000 (Thermo Fisher Scientific) according to the manufacturer's instructions.

### Immunocytochemistry

Cells were seeded at a density of 2 × 10^4^ cells per well in 96‐well plates. After experimental treatment such as compressive loading or transfection, cells were fixed with 4% paraformaldehyde for 10 min and then blocked/permeabilized with PBS containing 0.3% Triton X‐100 (FUJIFILM Wako Pure Chemical Corporation) and 5% goat serum (Gibco‐BRL) for 30 min at room temperature. Cells were then incubated with anti‐VNUT rabbit polyclonal antibody (1 : 100 dilution) [24], anti‐VNUT guinea pig polyclonal antibody (1 : 100 dilution, ABN83; Sigma‐Aldrich), or anti‐V5 antibody mouse monoclonal antibody (1 : 100 dilution, M167‐3, MBL) for 1 h at room temperature. Following incubation with an Alexa 488‐conjugated secondary antibody (1 : 1000 dilution; Thermo Fisher Scientific), cells were imaged with an ABZ‐9000 microscope (Keyence, Osaka, Japan). To visualize the cell nuclei, the cells were stained with DAPI (1 : 1000 dilution; Vector laboratories). To visualize the cytoskeleton, the cells were stained with Rhodamine Phalloidin (1 : 1000 dilution; Thermo Fisher Scientific). The fluorescence intensity per unit area of the cells was quantified using imagej (National Institutes of Health, Bethesda, MD, USA) [33] . All experiments were performed at least three independent times. All images were acquired at the same exposure and contrast settings, and representative images are shown.

### Measurement of ALP activity

Cells were seeded at a density of 3 × 10^5^ cells per well in 12‐well plates. After 7‐day treatment with osteoblast differentiation medium, cells were washed with PBS and lysed by freeze–thaw lysis in 0.1% Triton X (FUJIFILM Wako Pure Chemical Corporation). Cell lysates were then incubated with a substrate solution of 1.5 mm MgCl_2_ (pH 10), 50 mm NaHCO₃/Na₂CO₃, 1 mg·mL^−1^ p‐nitrophenyl phosphate, and absorbance (O.D.) was measured at 405 nm using a microplate reader (Bio‐Rad Laboratories, Inc., Hercules, CA, USA) [34].

### Short hairpin RNA (shRNA) gene silencing

Vectors expressing shRNAs targeting *Slc17a9* (sh‐Vnut, TRCN0000102020, TRCN0000102021, TRCN0000102022) and the nontargeted control shRNA (sh‐scr; NSHMC016) were purchased from Sigma‐Aldrich. For stable silencing of *Slc17a9*, lentiviral particles were prepared by transfecting HEK‐293T cells with shRNA vector, psPAX2 viral packaging vector (a gift from D. Trono; Addgene plasmid #12260), and pMD2. G viral envelope encoding vector (a gift from D. Trono; Addgene plasmid #12259), using Lipofectamine 2000 (Thermo Fisher Scientific). Lentivirus was collected 48 h post‐transfection and filtered using a 0.45‐μm filter (Merck KGaA). Viral infection of MC3T3‐E1 cells was performed with 8.0 μg·mL^−1^ hexadimethrine bromide for 24 h. Stable cells were then selected by culturing in the presence of 5.0 μg·mL^−1^ puromycin (FUJIFILM Wako Pure Chemical Corporation) for 3 days.

### siRNA knockdown

siRNA against *P2x7r* (Stealth siRNA, forward, ACGAAGUUAGGACACAGCAUCUUUG; reverse, CAAAGAUGCUGUGUCCUAACUUCGU) (Thermo Fisher Scientific) or *P2y2r* (Stealth siRNA, forward, CCCUGCCGCUGUUGGUUUAUUACUA; reverse, UAGUAAU AAACCAACAGCGGCAGGG) was transfected into MC3T3‐E1 cells using Lipofectamine RNAiMAX Transfection Reagent (Thermo Fisher Scientific) according to the manufacturer's protocol.

### Statistical analysis

Comparisons were made using unpaired *t*‐test, one‐way analysis of variance and using the Bonferroni method or Kruskal–Wallis method tests. The results are shown as the mean ± SD. The statistical significance is indicated as follows: *P < 0.05 and **P < 0.01.

## Results

### VNUT is expressed by osteoblasts

**Fig. 1:**
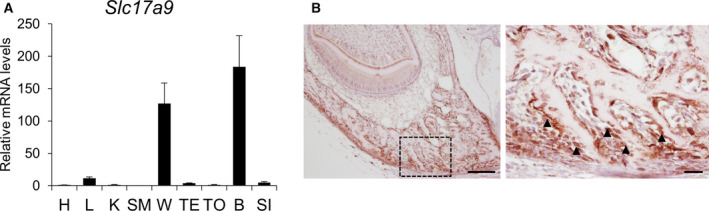
VNUT is expressed in osteoblasts. (A) The mRNA levels of *Slc17a9* in various murine tissues were determined by qPCR. (B) Mandibular bone from 3‐day‐old C57BL/6J mice was immunostained with rabbit anti‐VNUT antibody. The boxed areas in the left panel are shown as magnified images in the right panel. Scale bars indicate 100 μm (left panel) and 20 μm (right panel), respectively. Arrowheads in the right panel indicate VNUT‐positive osteoblasts. Data are expressed as the mean ± SD (*n* = 3). heart, H; liver, L; kidney, K; skeletal muscle, SM; white adipose tissue, W; testis, TE; tongue, TO; bone, B; small intestine, SI.

Expression of *Slc17a9* in various murine tissues was examined by qPCR. mRNA levels of *Slc17a9* were particularly high in bone and white adipose tissue (Fig. 1A). Immunohistochemical analysis revealed that VNUT was expressed in osteoblasts within alveolar bone (Figs 1B and S1A). The specificity of antibody against VNUT was confirmed using negative and positive controls (Fig. S1B‐D).

### Mechanical loading increases VNUT expression levels

**Fig. 2:**
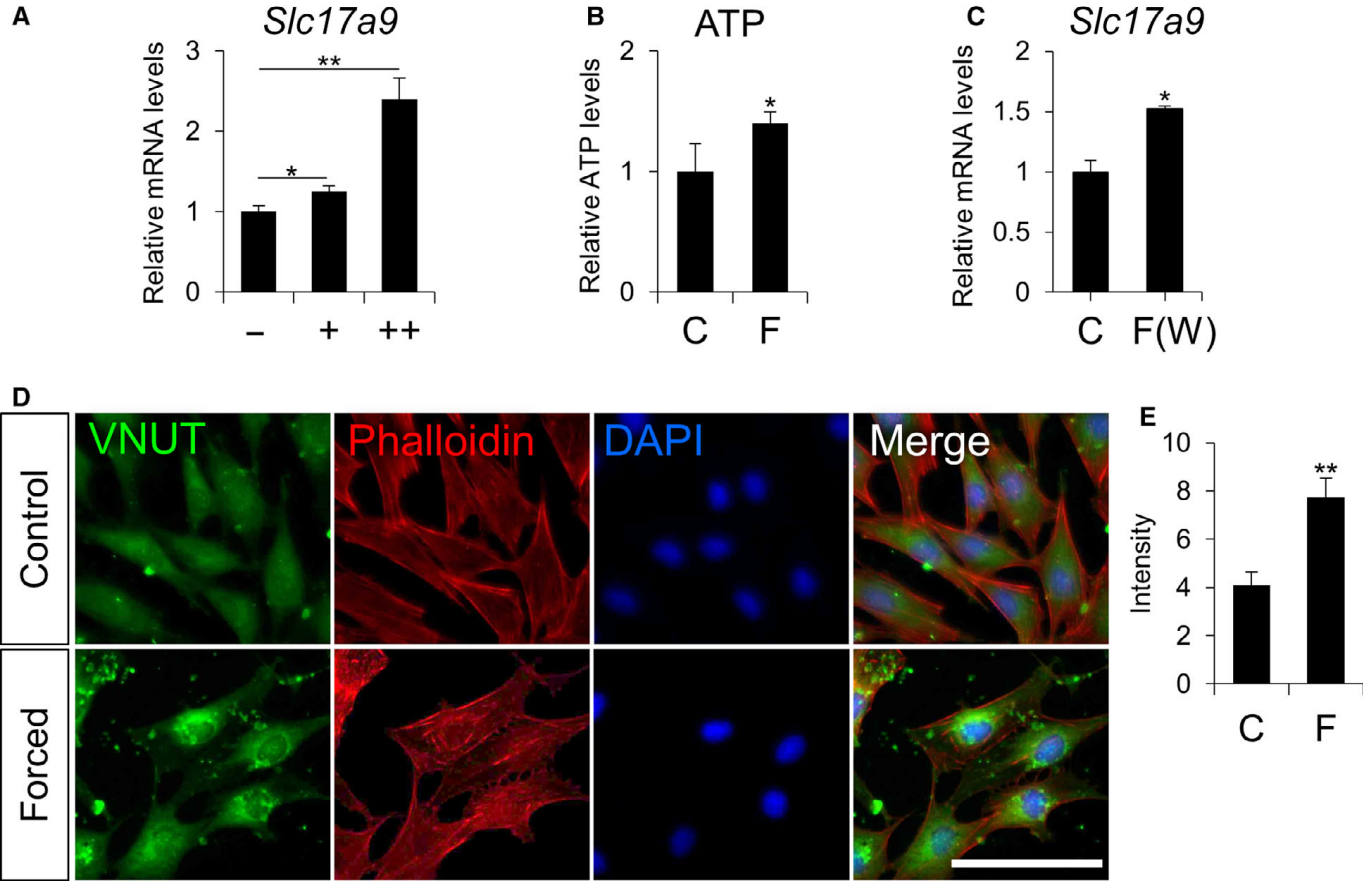
Compressive force stimulates expression of *Slc17a9*. (A) mRNA levels of *Slc17a9* in MC3T3‐E1 cell exposed to 0, 4.4 × 10^−2^, or 8.8 × 10^−2^ N·cm^−2^ of compressive force by centrifuge for 12 h. (B) Extracellular ATP levels in MC3T3‐E1 cell exposed to 8.8 × 10^−2^ N·cm^−2^ of compressive force by centrifuge for 12 h. (C) mRNA levels of *Slc17a9* in MC3T3‐E1 cells exposed to 8.8 × 10^−2^ N·cm^−2^ compressive force by weights for 12 h. (D) MC3T3‐E1 cells treated with or without 8.8 × 10^−2^ N·cm^−2^ compressive force by centrifuge for 12 h were stained with guinea pig anti‐VNUT antibody, rhodamine phalloidin, or DAPI. Scale bars indicate 100 μm. (E) The fluorescence intensity of the VNUT staining shown in (D) was quantified by imagej. control, C; compressive force by centrifuge, F; compressive force by weights, F(W). Data are expressed as the mean ± SD (*n* = 3). Statistical analysis was performed with one‐way analysis of variance followed by Bonferroni method (A) and unpaired *t*‐test (B, C, E). *P < 0.05 or **P < 0.01 versus control.

Since mechanical force stimulates ATP release in osteoblasts, we next examined whether compressive force on MC3T3‐E1 cells affects *Slc17a9* expression. 4.4 × 10^−2^ or 8.8 × 10^−2^ N·cm^−2^ loading for 12 h upregulated the mRNA levels of *Slc17a9* in load‐dependent manner in MC3T3‐E1 cells (Fig. 2A). Under these conditions, there was no obvious cytotoxicity to cells as assessed by LDH assay (data not shown). The increase in *Slc17a9* expression after 12 h of 8.8 × 10^−2^ N·cm^−2^ compressive force was correlated with an increase in extracellular ATP (Fig. 2B). To further assess the role of mechanical loading on *Slc17a9* expression, we used an alternate weight‐induced mechanical loading model to apply compressive force to MC3T3‐E1 cells. 8.8 × 10^−2^ N·cm^−2^ of force for 12 h led to a significant upregulation of *Slc17a9* mRNA levels (Fig. 2C). Furthermore, immunostaining showed that compressive force increased the fluorescence intensity of VNUT staining (Fig. 2D,E). As with the immunohistochemical staining, the specificity of antibody against VNUT was confirmed using negative/positive control experiments (Fig. S2).

### Mechanical loading inhibits osteoblast differentiation

**Fig. 3:**
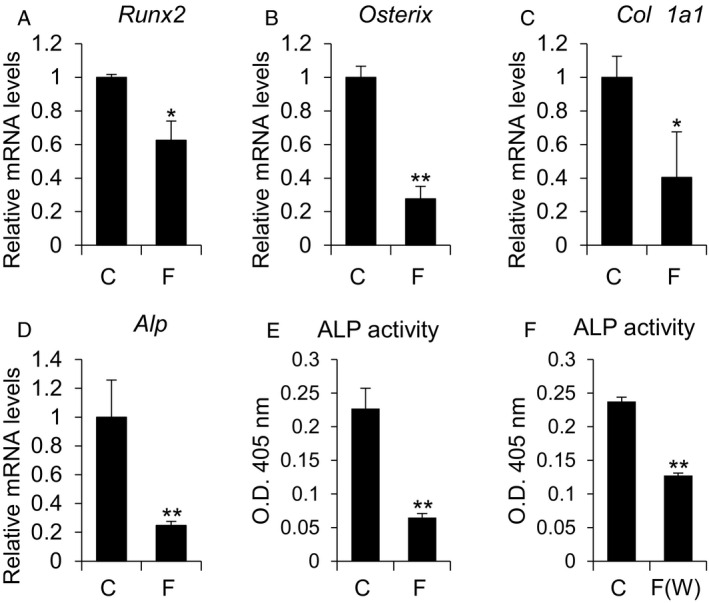
Compressive force suppresses osteoblast differentiation. (A–E) MC3T3‐E1 cells were treated with osteoblast differentiation medium for 7 days after the application of compressive force (8.8 × 10^−2^ N·cm^−2^, 12 h), and mRNA levels of the indicated genes were determined by qPCR. (E) ALP activity in cells treated as in A‐E (F) ALP activity in MC3T3‐E1 cells treated with osteoblast differentiation medium for 7 days after the application of 8.8 × 10^−2^ N·cm^−2^ compressive force by weight for 12 h. control, C; compressive force by centrifuge, F; compressive force by weights, F(W). Data are expressed as the mean ± SD (*n* = 3). Statistical analysis was performed with unpaired *t*‐test. *P < 0.05 or **P < 0.01 versus control.

We next examined the effect of mechanical loading‐induced VNUT on osteoblast differentiation. MC3T3‐E1 cells were mechanically loaded by centrifugation and then stimulated to differentiate by the addition of ascorbic acid and β‐glycerophosphate. As shown in Fig. 3A‐D, compressive force suppressed the expression of osteoblast differentiation markers such as *Runx2* (Fig. 3A), *Osterix* (Fig. 3B), *Collagen 1a1* (Fig. 3C), and *Alp* (Fig. 3D). In addition, ALP activity, an indicator of osteoblast function, was also decreased following compressive force applied by centrifugation (Fig. 3E) or by weight loading (Fig. 3F).

### VNUT inhibits osteoblast differentiation

**Fig. 4:**
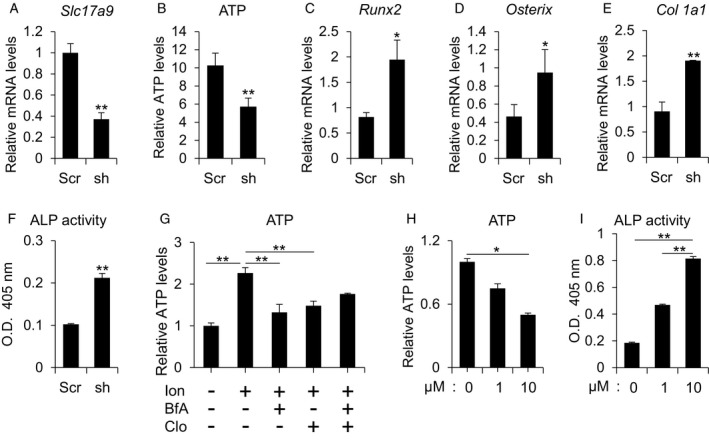
Knockdown or inhibition of VNUT stimulates osteoblast differentiation. (A) mRNA levels of *Slc17a9* in MC3T3‐E1 cells stably expressing control scrambled shRNA or shRNA against murine *Slc17a9*. (B) Extracellular ATP in MC3T3‐E1 cells stably expressing control scrambled shRNA or shRNA against murine *Slc17a9* exposed to compressive force by centrifuge (8.8 × 10^−2^ N·cm^−2^, 12 h). (C‐E) MC3T3‐E1 cells stably expressing control scrambled shRNA or shRNA against murine *Slc17a9* were treated with osteoblast differentiation medium for 7 days after the application of compressive force by centrifuge (8.8 × 10^−2^ N·cm^−2^, 12 h) and mRNA levels of the indicated osteoblast marker genes measured by qPCR or (F) ALP activity determined. (G) Extracellular ATP levels in MC3T3‐E1 cells treated with 1 μm ionomycin, 10 μm brefeldin A, or 10 μm clodronate for 30 min. (H) MC3T3‐E1 cells were exposed to compressive force by centrifugation (8.8 × 10^−2^ N·cm^−2^, 12 h) and treated with 0, 1.0, or 10 μm clodronate after which extracellular ATP was measured. (I) MC3T3‐E1 cells were treated with 0, 1.0, or 10 μm clodronate along with osteoblast differentiation medium, and ALP activity was measured 7 days after the application of compressive force by centrifuge (8.8 × 10^−2^ N·cm^−2^, 12 h). scrambled shRNA, Scr; shRNA against murine *Slc17a9*, sh; ionomycin, Ion; brefeldin A, BfA; clodronate, Clo. Data are expressed as the mean ± SD (*n* = 3). Statistical analysis was performed with unpaired *t*‐test (A‐F) and one‐way analysis of variance followed by Bonferroni method (G and H) or Kruskal–Wallis method test (I). *P < 0.05 or **P < 0.01 versus scramble (A–F) or control (H and I).

To determine whether VNUT affects osteoblast differentiation, we stably knocked down *Slc17a9* in MC3T3‐E1 cells using shRNA (Fig. 4A). Extracellular ATP levels were decreased in *Slc17a9*‐knockdown cells after mechanical loading (Fig. 4B). In the absence of mechanical loading, extracellular ATP levels were unchanged (Fig. S3). In response to osteogenic medium, *Slc17a9*‐knockdown cells displayed enhanced osteoblast differentiation, as indicated by a higher expression of osteoblast differentiation marker mRNA expression (Fig. 4C–E) and increased ALP activity (Fig. 4F) under the mechanical loading condition. In order to confirm the role of VNUT and exocytosis in ATP release, we next treated cells with clodronate, a VNUT inhibitor [35], in combination with ionomycin and brefeldin A. Ionomycin is a selective calcium ionophore that rapidly induces an increase in intracellular calcium leading to ATP release. Brefeldin A is an inhibitor of vesicular exocytosis. As shown in Fig. 4G, clodronate and brefeldin A both suppressed release of ATP from MC3T3‐E1 cells suggesting that ATP release is dependent on VNUT activity and exocytosis. Clodronate decreased extracellular ATP levels in a concentration‐dependent manner (Fig. 4H) and increased ALP activity in response to mechanically loading (Fig. 4I). These observations were independent of changes in cell number since clodronate did not affect proliferation of MC3T3‐E1 cells (Fig. S4).

### Compressive force stimulates P2 receptor expression and regulates osteoblast differentiation via P2 receptors

**Fig. 5:**
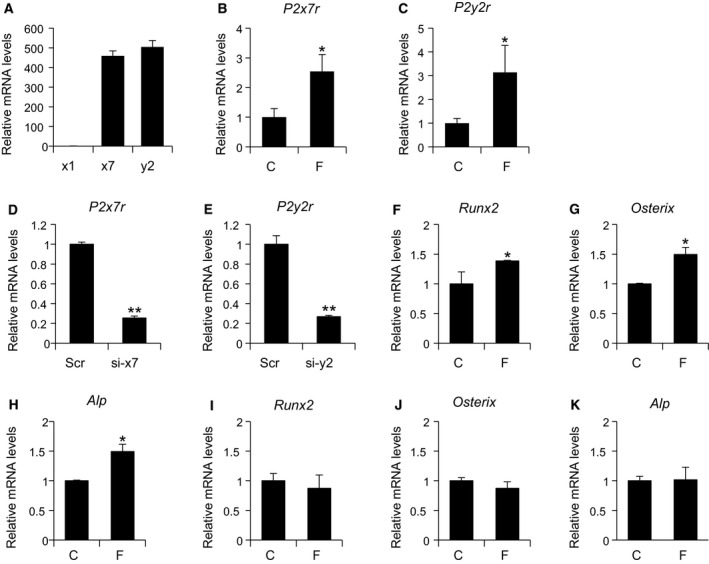
P2X7 and/or P2Y2 receptors are involved in the suppressive effect of osteoblast differentiation by compressive force. (A) mRNA levels of *P2x1r*, *P2x7r*, and *P2y2r* in proliferating MC3T3‐E1 cells. (B and C) mRNA levels of *P2x7r* and *P2y2r* were increased after the application of compressive force by centrifugation (8.8 × 10^−2^ N·cm^−2^, 12 h). (D) Cells were transfected with scramble siRNA or siRNA against *P2x7r* and mRNA levels of *P2x7r* determined 24 h after transfection. (E) Cells were transfected with scramble siRNA or siRNA against *P2y2r* and mRNA levels of *P2y2r* determined 24 h after transfection. (F‐H) Cells transfected with siRNA against *P2x7r* were treated with osteoblast differentiation medium and mRNA levels of the indicated osteoblast marker genes determined 7 days after application of compressive force by centrifuge (8.8 × 10^−2^ N·cm^−2^, 12 h). (I–K) Cells transfected with siRNA against *P2y2r* were treated with osteoblast differentiation medium and mRNA levels of the indicated osteoblast marker genes determined 7 days after application of compressive force by centrifuge (8.8 × 10^−2^ N·cm^−2^, 12 h). *P2x1r*, x1; *P2x7r*, x7; *P2y2r*, y2; control, C; compressive force by centrifuge, F; scrambled siRNA, Scr; siRNA against *P2x7r*, si‐x7; siRNA against *P2y2r*, si‐y2. Data are expressed as the mean ± SD (*n* = 3). Statistical analysis was performed with unpaired *t*‐test. *P < 0.05 or **P < 0.01 versus control (B, C, F–H, I–K) or scramble (D and E).

To explore potential mechanisms downstream of mechanical loading‐induced VNUT, we next sought to determine the role of P2 receptors. Among the P2 receptor family, P2X1R [14], P2X7R [12, 13, 15, 18], and P2Y2R [11, 16, 17] have been reported to be involved in osteoblastogenesis. *P2x7r* and *P2y2r* were highly expressed in MC3T3‐E1 cells relative to *P2x1r* (Fig. 5A). Expression levels of *P2x7r* (Fig. 5B) and *P2y2r* (Fig. 5C) were increased by compressive loading. Transfection of siRNA against *P2x7r* (Fig. 5D) or *P2y2r* (Fig. 5E) significantly reduced the expression levels of *P2x7r* and *P2y2r* in MC3T3‐E1 cells. Knockdown of *P2x7r* and *P2y2r* canceled the suppression of osteoblast differentiation caused by mechanical loading (Fig. 5F‐H,I‐K).

## Discussion

**Fig. 6:**
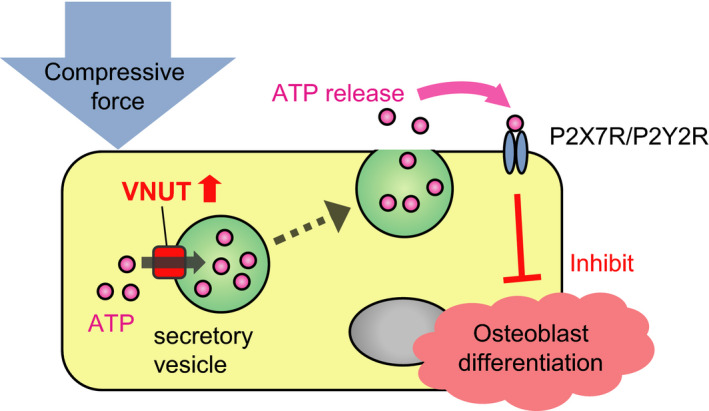
Model for the suppressive effect of compressive force on osteoblast differentiation. Compressive force increases extracellular ATP via induction of VNUT. Consequently, ATP regulates osteoblast differentiation through the P2X7 and/or P2Y2 receptor.

The data presented in this study reveal a role for VNUT in osteoblast differentiation and function. We show that VNUT is expressed in bone and in osteoblasts, and we present evidence that VNUT inhibits osteoblast differentiation in response to compressive force. Furthermore, we report that VNUT actions are linked to ATP release and P2X7R and/or P2Y2R activity (Fig. 6).

Our previous work on VNUT showed its importance in the function of odontoblasts, a mineral‐forming cell type similar in many ways to osteoblasts [24]. In addition, we have found VNUT expression in periodontal ligament cells [25] and dental pulp cells [24]. VNUT expression has also been observed in various other tissues such as biliary epithelial cells [36] and intestinal L cells [37]. Thus, VNUT is a largely ubiquitous nucleotide transporter, but its expression may be specially adapted in each cell type to metabolic demands and requirements for ATP after specific stimuli. More specifically, in osteoblasts we observed that VNUT expression is regulated by mechanical force.

Regulation of VNUT by mechanical stimuli is consistent with our previous observations in human periodontal ligament cells [25] where VNUT expression increased with the application of compressive force. Other reports suggest that hyperosmotic stress increases VNUT expression in human corneal and conjunctival epithelial cell lines and could be involved in ATP release at the ocular surface [38]. In dental pulp cells however, VNUT expression was regulated by heat stimulation [24]. In our preliminary data, heat stimulation also increased VNUT expression in MC3T3‐E1 cells (data not shown). It has been reported that heat stress controls osteoblast differentiation [39, 40, 41], so it is also possible that VNUT is involved in the effects of heat stimulation on osteoblast differentiation. In gastric epithelial cells, VNUT is involved in ATP release induced by activation of transient receptor vanilloid 4, a nonselective cation channel activated by mechanical stimulation, osmotic pressure, heat, or chemicals [26]. Our observation that VNUT expression correlates with extracellular ATP levels is consistent with findings in odontoblasts and periodontal ligament cells as well [24, 25].

Here, in this study, we report that VNUT inhibits osteoblast differentiation. Given that ATP has also been linked to osteoblast differentiation [11, 12, 13, 14, 15, 16, 17, 18], it is easy to speculate that VNUT‐induced ATP release contributes to the inhibition of differentiation. Indeed, treatment of MC3T3‐E1 cells with clodronate, an inhibitor of vesicular ATP release, also enhanced osteoblast differentiation. VNUT decreases neurogenesis in N2a murine neuroblastoma cells [42]. Both neuroblasts and osteoblasts belong to mesenchymal lineage cells. We also noted that VNUT was highly expressed in white adipose tissue (Fig. 1A). Perhaps VNUT may regulate the differentiation of other mesenchymal lineage cells, such as adipocytes as well.

In this study, the application of compressive force by centrifugal force suppressed osteoblast differentiation. Kariya et al found that applying tension force to MC3T3‐E1 cells increased Runx2 and Osterix expression, extracellular matrix proteins, and ALP activity and promoted calcium mineral formation [12]. A study of low‐intensity pulsed ultrasound stimulation of MC3T3‐E1 cells reported increased expression of Runx2 and Osterix [13]. On the other hand, in a study in which osteoblasts collected from mouse mandibles were subjected to hydrostatic pressure, the production of inflammatory cytokines and RANKL, an activator of bone remodeling, was increased [43]. The inconsistency in these results may be due to the fact that the cellular response to mechanical load differs depending on the type, magnitude, time, and cell used.

Adenosine triphosphate induces various physiological responses through the activation of P2 receptors. P2 receptors are divided into the P2Y family of G protein‐coupled receptors and the P2X family of ligand‐gated cation channels [44]. Osteoblasts have been reported to express both P2X and P2Y receptors [45]. Our results showed that compressive force on MC3T3‐E1 cells increased ATP release and increased expression of *P2x7r* and *P2y2r*. Knockdown of *P2x7r* and *P2y2r* canceled the suppression of osteoblast differentiation caused by the application of compressive force. These results support the inhibitory effects of P2X7R and P2Y2R on bone formation that have been previously reported [15, 16, 17, 18].

Taken together, our findings suggest that compressive‐induced VNUT regulates osteoblast differentiation, at least in part, via the extracellular ATP‐P2X7R and/or P2Y2R pathway. Unfortunately, we could not reveal the downstream effector(s) of this extracellular ATP‐P2X7R and/or P2Y2R pathway. It has been reported that P2X7R or P2Y2R are related with osteogenic pathways such as WNT/β‐catenin signaling [15] or ERK1/2 signaling [11]. Thus, mechanism of VNUT function may involve crosstalk with such signaling pathways. Further research to explore the mechanisms and implications of these observations may help the development of new therapeutics for osteoporosis, bone‐related diseases, and orthodontic treatment in an aging society.

## Conflict of interest

The authors declare no conflict of interest.

## Author contributions

AI, TS, MN, TM, and SK performed the experiments. AI, KN‐K, KK‐G, MM, TS, MI‐S, KY, MN, TM, YT‐S, KM, SK, and TK reviewed the intermediate draft. AI and KN‐K designed the study. AI, KN‐K, SK, and TK performed the literature review, prepared the initial and final versions of the article, and submitted the document.

## Supporting information


**Fig. S1.** Immunohistochemistry with VNUT antibodies. Mandibular bone from 3‐days‐old C57BL/6J mice was immunostained with (A) VNUT antibody (Guinea pig), (B) PBS alone as a negative control, (C) VNUT antibody (Rabbit) pre‐absorbed with VNUT peptide antigen as a negative control for the Rabbit VNUT antibody, or (D) VNUT antibody (Rabbit). Arrowheads in the (A) panel and (D) left panel indicate VNUT positive osteoblasts and odontoblasts respectively. Odontoblasts, used as a positive control, have previously been shown to highly express VNUT [24]. Boxed areas in the (C, D) left panel are shown as magnified images in the right panel. Scale bars indicate 20 µm (A, B, C right panel, D right panel) and 100 µm (C right panel, D right panel) respectively.

**Fig. S2.** Immunocytochemistry with VNUT antibodies. (A) MC3T3‐E1 cells are immunostained with Rabbit VNUT antibody preabsorbed with VNUT blocking antigen peptide (Control) or VNUT antibody (Rabbit). (B) MC3T3‐E1 cells stably expressing control scrambled shRNA or shRNA against murine *Slc17a9* were stained with VNUT antibody (Rabbit), rhodamine phalloidin, or DAPI. (C) MC3T3‐E1 cells were transfected with V5‐tagged VNUT and immunostained with VNUT antibody (Rabbit or Guinea pig), and anti‐V5 antibody and DAPI. Scale bars indicate 100 µm, scrambled shRNA, Scr; shRNA against murine *Slc17a9*, Sh.

**Fig. S3.** Knock down of VNUT does not alter extracellular ATP levels in the absence of mechanical force. Extracellular ATP levels from MC3T3‐E1 cells stably expressing control scrambled shRNA or shRNA against *Slc17a9*. Scrambled shRNA, Scr; shRNA against murine *Slc17a9*, sh. Data are expressed as the mean ± SD (*n* = 3). Statistical analysis was performed with unpaired *t*‐test. *P < 0.05 or **P < 0.01 versus control.

**Fig. S4.** Inhibition of Vnut function does not affect cell proliferation of MC3T3‐E1 cells. MC3T3‐E1 cells were plated into 96‐well plates and incubated with 0, 1.0, or 10 μM clodronate. Cell proliferation was assessed on day 1, 2, or 3 using a Cell Counting Kit‐8 (DOJINDO).

## Data Availability

Raw data are available from the corresponding author upon reasonable request.
