# Supplementary figures and images for: VNUT/SLC17A9, a vesicular nucleotide transporter, regulates osteoblast differentiation
Source: FEBS Open Bio. 2020 Jul 12;10(8):1612–23. doi: 10.1002/2211-5463.12918 (PMC7396442; doi:10.1002/2211-5463.12918)

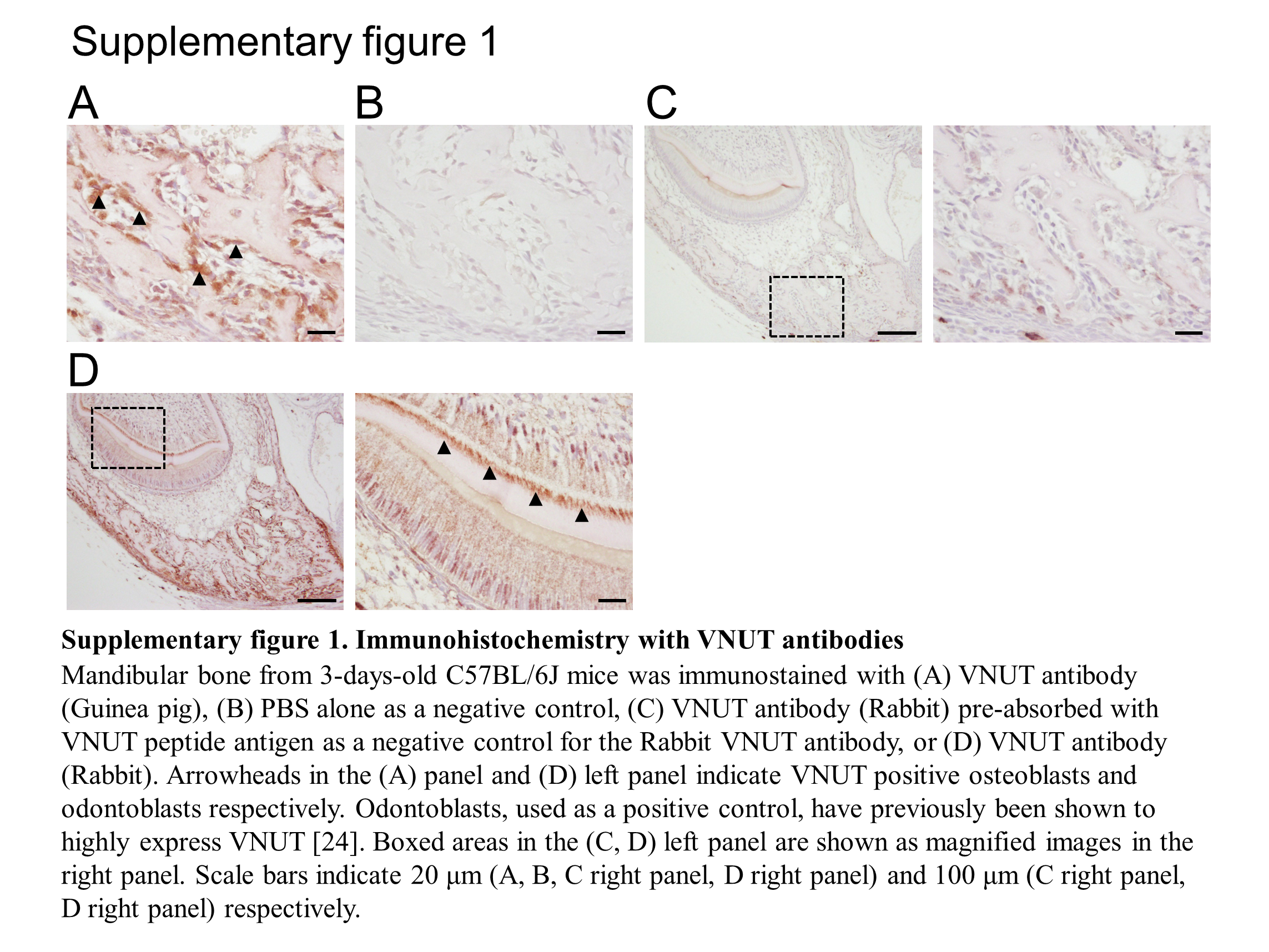

Supplement: Supplementary file 1 — Fig. S1. Immunohistochemistry with VNUT antibodies. Mandibular bone from 3‐days‐old C57BL/6J mice was immunostained with (A) VNUT antibody (Guinea pig), (B) PBS alone as a negative control, (C) VNUT antibody (Rabbit) pre‐absorbed with VNUT peptide antigen as a negative control for the Rabbit VNUT antibody, or (D) VNUT antibody (Rabbit). Arrowheads in the (A) panel and (D) left panel indicate VNUT positive osteoblasts and odontoblasts respectively. Odontoblasts, used as a positive control, have previously been shown to highly express VNUT [24]. Boxed areas in the (C, D) left panel are shown as magnified images in the right panel. Scale bars indicate 20 µm (A, B, C right panel, D right panel) and 100 µm (C right panel, D right panel) respectively. [file FEB4-10-1612-s001.PNG]

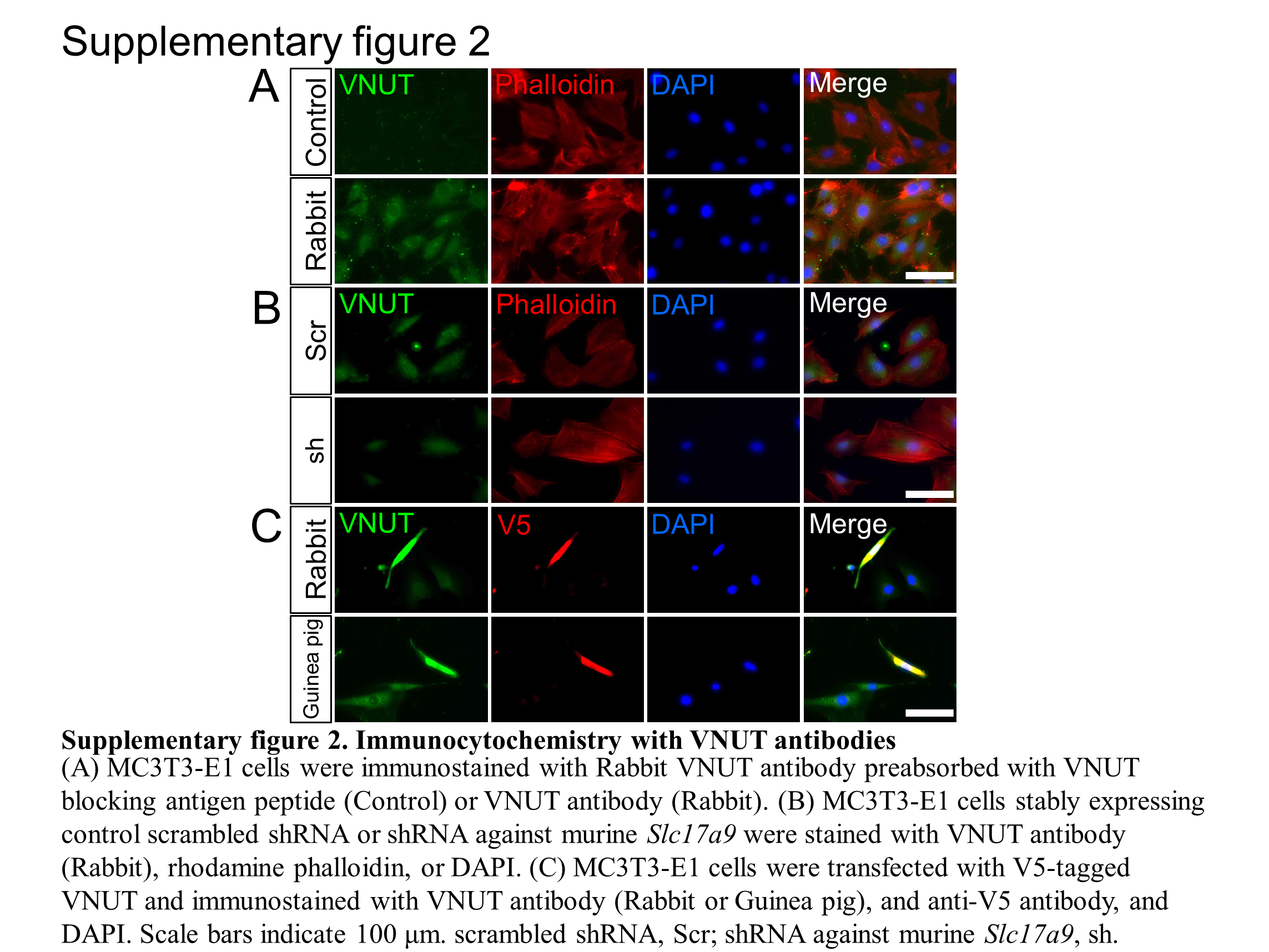

Supplement: Supplementary file 2 — Fig. S2. Immunocytochemistry with VNUT antibodies. (A) MC3T3‐E1 cells are immunostained with Rabbit VNUT antibody preabsorbed with VNUT blocking antigen peptide (Control) or VNUT antibody (Rabbit). (B) MC3T3‐E1 cells stably expressing control scrambled shRNA or shRNA against murine Slc17a9 were stained with VNUT antibody (Rabbit), rhodamine phalloidin, or DAPI. (C) MC3T3‐E1 cells were transfected with V5‐tagged VNUT and immunostained with VNUT antibody (Rabbit or Guinea pig), and anti‐V5 antibody and DAPI. Scale bars indicate 100 µm, scrambled shRNA, Scr; shRNA against murine Slc17a9, Sh. [file FEB4-10-1612-s002.PNG]
