# Supplementary material for: VNUT/SLC17A9, a vesicular nucleotide transporter, regulates osteoblast differentiation
Source: FEBS Open Bio. 2020 Jul 12;10(8):1612–23. doi: 10.1002/2211-5463.12918 (PMC7396442; doi:10.1002/2211-5463.12918)
Supplement: Supplementary file 3 — Fig. S3. Knock down of VNUT does not alter extracellular ATP levels in the absence of mechanical force. Extracellular ATP levels from MC3T3‐E1 cells stably expressing control scrambled shRNA or shRNA against Slc17a9. Scrambled shRNA, Scr; shRNA against murine Slc17a9, sh. Data are expressed as the mean ± SD (n = 3). Statistical analysis was performed with unpaired t‐test. *P < 0.05 or **P < 0.01 versus control. [file FEB4-10-1612-s003.pdf]

Supplementary figure 3

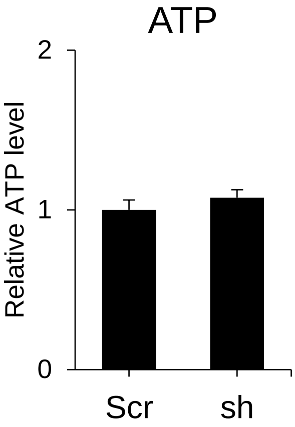

Supplementary figure 3. Knock down of VNUT does not alter extracellular ATP levels in the absence of mechanical force

Extracellular ATP levels from MC3T3-E1 cells stably expressing control scrambled shRNA or shRNA against Slc17a9. Scrambled shRNA, Scr; shRNA against murine Slc17a9, sh. Data are expressed as the mean ± SD (n = 3). Statistical analysis was performed with unpaired t-test. *, $p < 0.05$ or **, $p < 0.01$ versus control.
