# Supplementary material for: VNUT/SLC17A9, a vesicular nucleotide transporter, regulates osteoblast differentiation
Source: FEBS Open Bio. 2020 Jul 12;10(8):1612–23. doi: 10.1002/2211-5463.12918 (PMC7396442; doi:10.1002/2211-5463.12918)
Supplement: Supplementary file 4 — Fig. S4. Inhibition of Vnut function does not affect cell proliferation of MC3T3‐E1 cells. MC3T3‐E1 cells were plated into 96‐well plates and incubated with 0, 1.0, or 10 μM clodronate. Cell proliferation was assessed on day 1, 2, or 3 using a Cell Counting Kit‐8 (DOJINDO). [file FEB4-10-1612-s004.pdf]

Supplementary figure 4

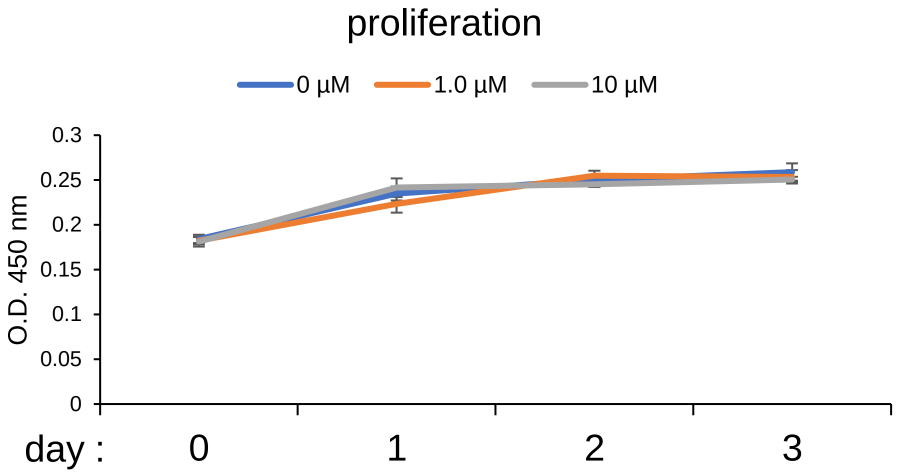

Supplementary figure 4. Inhibition of Vnut function does not affect cell proliferation of MC3T3-E1 cells

MC3T3-E1 cells were plated into 96-well plates and incubated with 0, 1.0, or 10 µM clodronate. Cell proliferation was assessed on day 1, 2, or 3 using a Cell Counting Kit-8 (DOJINDO).
